# Functional abdominal pain disorders in children in southern Anhui Province, China are related to academic stress rather than academic performance

**DOI:** 10.1186/s12887-023-04154-3

**Published:** 2023-06-29

**Authors:** Xiaoshuang Bao, Wenchao Yu, Ziyan Chu, Jie Gao, Meimei Zhou, Yong Gu

**Affiliations:** 1 Department of Paediatrics, Graduate School, Wannan Medical College, Wuhu, Anhui China; 2 Department of Paediatrics, Yijishan Hospital of Wannan Medical College, No. 2, Zheshan Road, Wuhu, 241000 Anhui Province People’s Republic of China

**Keywords:** Academic stress, Children, Functional abdominal pain disorders, Rome IV criteria

## Abstract

**Background:**

Functional abdominal pain disorders (FAPDs) are one of the most common gastrointestinal disorders in children. The aim of this study was to investigate the prevalence of FAPDs in children in southern Anhui Province, China and their association with academic stress.

**Methods:**

In this cross-sectional survey, we randomly selected children aged 6–17 years from 11 public schools in southern Anhui Province. FAPDs were diagnosed according to the Rome IV criteria, and a custom-designed questionnaire was used to investigate the association between academic stress and FAPDs in children.

**Results:**

A total of 2,344 children aged 6–17 years were enrolled. The mean age was 12.4 ± 3.0 years. Of these children, 335 (14.3%) were diagnosed with FAPDs according to the Rome IV criteria. Among the children with FAPDs, 156 (46.6%) were boys, and 179 (53.4%) were girls. The prevalence was higher in girls than in boys. The most common disorder was irritable bowel syndrome (IBS) (n = 182 (7.8%)). Other types of FAPDs included functional abdominal pain–not otherwise specified (FAPNOS) (n = 70 (3.0%)), functional dyspepsia (FD) (n = 55 (2.3%)), and abdominal migraine (AM) (n = 28 (1.2%)). Academic stress, not meeting parental expectations, poor relationships with parents, and sleep disturbances were independent risk factors for FAPDs in children; academic performance was not associated with the development of FAPDs.

**Conclusion:**

There was a high prevalence of FAPDs among children in southern Anhui Province, China, and IBS was the most common subtype of functional abdominal pain. Academic stress, rather than academic performance, was associated with FAPDs in children.

## Background

Functional abdominal pain disorders (FAPDs) are a group of disorders involving significant and recurrent symptoms of gastrointestinal discomfort without corresponding abnormalities on instrumental and laboratory tests. These symptoms are also difficult to explain in terms of structural or biochemical abnormalities of the digestive system [1]. FAPDs are chronic disorders and the repeated attacks not only reduce quality of life of patients but also increase the risks of anxiety, depression, and school absenteeism and reduce academic performance [2]. FAPDs are common paediatric diseases and are diagnosed with the symptom-based Rome criteria. According to Rome IV criteria, FAPDs are defined as disorders with symptoms lasting 4 days per month over at least 2 months [1]. Compared to the Rome III criteria [3], the Rome IV criteria allow clinicians to diagnose functional gastrointestinal disorders (FGIDs) selectively with or without clinical testing. In these newer diagnostic criteria, the item “No evidence of inflammatory, anatomical, metabolic or neoplastic disease to explain the patient’s symptoms” was replaced with “The patient’s symptoms cannot be attributed to other medical conditions after appropriate medical evaluation” [4–6]. FAPDs are divided into four subtypes according to the Rome IV criteria: functional dyspepsia (FD), irritable bowel syndrome (IBS), abdominal migraine (AM) and functional abdominal pain–not otherwise specified (FAPNOS).

The pathogenesis of FAPDs has not been elucidated. According to recent studies, it may be associated with abnormal gastrointestinal motility, decreased visceral nociceptive thresholds, abnormal brain-gut interactions, psychosocial disorders, and immune activation [7–9]. The appearance and disappearance of symptoms of FAPDs are often related to psychological and social factors that cannot be fully explained by the biological model of pathophysiology alone. With the transformation of the medical model from a “biomedical model” to a “biopsychosocial” model, more attention to the relationship between external environmental stress and FAPDs is needed. Childhood and adolescence are important periods of physical and mental development, and the psychological stress caused by the gap between the ideal and reality can lead to the occurrence of FAPDs in these periods. Usually, FAPDs do not affect the growth and development of children, but severe cases can exhibit nausea, vomiting and malnutrition. Moreover, because the disease is chronic and has repeated attacks, it affects the academic performance and quality of life of children. It can even lead to mental health issues in children. Some parents of children with FAPDs have insufficient understanding of the disease, and repeated examinations and prescriptions in the outpatient department over an extended period of time are common; this not only affects the quality of life of children but also wastes medical resources [10]. At present, the prevalence of FAPDs among children in China is increasing each year, and the risk factors for this disease remain unclear. In this study, we investigated the prevalence of FAPDs in children in southern Anhui Province, China, and analysed the relationship between academic stress exposure and FAPDs to determine a scientific basis for the prevention and treatment of the disease.

## Methods

### Study design and subjects

This cross-sectional study was conducted in 11 public primary and secondary schools in southern Anhui, China. A total of 2,344 students completed the interview between September 2022 and November 2022.

Children aged 6 to 17 years were included; those unwilling to participate in the study, ≥ 18 years or < 6 years of age, or with incomplete data were excluded. We also excluded patients with the following alarm symptoms: persistent right upper and lower abdominal pain, dysphagia, persistent vomiting, gastrointestinal bleeding, nocturnal diarrhoea, arthralgia, perianal abscess or haemorrhoids, uncontrolled weight loss, growth retardation, unexplained fever, rash, or recurrent oral ulcers.

### Data collection

Relevant questionnaires were completed according to the Rome IV criteria. After obtaining consent from the school and parents, all participating children were contacted, and all face-to-face interviews were conducted by trained investigators. Basic demographic data collected included their name, sex, age, height, weight, and only child status; presence of postprandial fullness, early satiation, epigastric pain or burning sensation; location and frequency of abdominal pain; duration of abdominal pain; stool condition (normal, diarrhoea, constipation, or alternating diarrhoea and constipation); relationship between abdominal pain and defecation; symptoms accompanying episodes of abdominal pain; presence of academic stress; academic performance; academic performance compared to parental expectations; sleep disorders; and parental relationship quality.

The academic stress was measured based on a single indicator from the study ‘Health Behaviour in School-Aged Children (HBSC)’. All the children were asked to answer how stressed they felt due to the schoolwork they must do. The response scale ranged from 1 to 4 (1 = not at all; 2 = a little; 3 = some; and 4 = a lot). Children who scored 3–4 are considered to have academic stress, while those who scored 1–2 are considered to have no academic stress [11, 12].

The study was approved by the Ethics Committee of Yijishan Hospital, and all guardians of children included in the study signed an informed consent form indicating voluntary consent to participate in the study.

### Diagnosis

According to the Rome IV criteria [13], FAPDs are classified into four subtypes: FD, IBS, AM, and FAPNOS.

### Statistical analysis

Statistical analyses were conducted using SPSS 26.0 software (IBM Corp., Armonk New York, USA) and R software (version 4.2.2). Categorical variables are reported as counts and percentages and were evaluated using chi-square tests or Fisher’s exact tests as appropriate. Continuous variables are reported as the mean ± SD and were evaluated using a *t* test or the Kruskal–Wallis test as appropriate. All variables were explored by univariate analysis, and variables with *P* values < 0.05 in univariate analysis were entered into the multivariate logistic regression analysis. Data were expressed as ORs and 95% confidence intervals (CIs). Statistical significance was defined as a result for which *P* < 0.05. A nomogram was constructed based on the logistic regression results. Discrimination was evaluated using the area under the receiver-operating characteristic (ROC) curve, and calibration was evaluated using the calibration curve and the Hosmer–Lemeshow test.

## Results

### Baseline patient characteristics

**Fig. 1:**
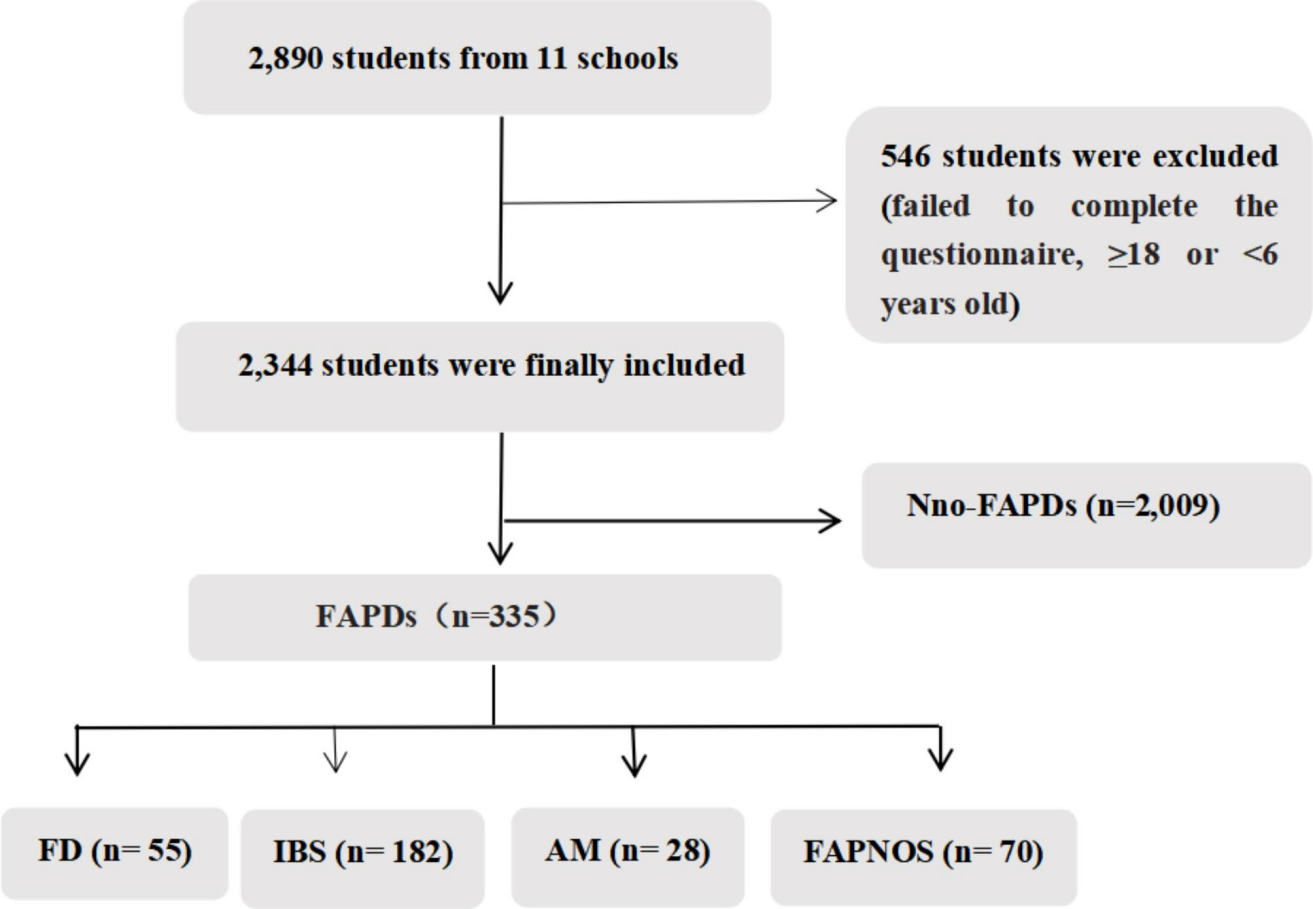
Flowchart of participant recruitment and group membership

We randomly selected 2,890 students from 11 public primary and secondary schools in southern Anhui between September 2022 and November 2022; of these students, 546 were excluded because they failed to complete the questionnaire or were ≥ 18 or < 6 years old (Fig. 1). A total of 2,344 questionnaires were successfully collected from students with an age range of 6–17 years and a mean age of 12.4 ± 3.0 years. Participants included 1,244 boys (53.1%) and 1,100 girls (46.9%), with 1,171 only children (49.9%) and 1,173 children with siblings (50.1%).

### Prevalence of FAPDs in children in southern Anhui

**Fig. 2:**
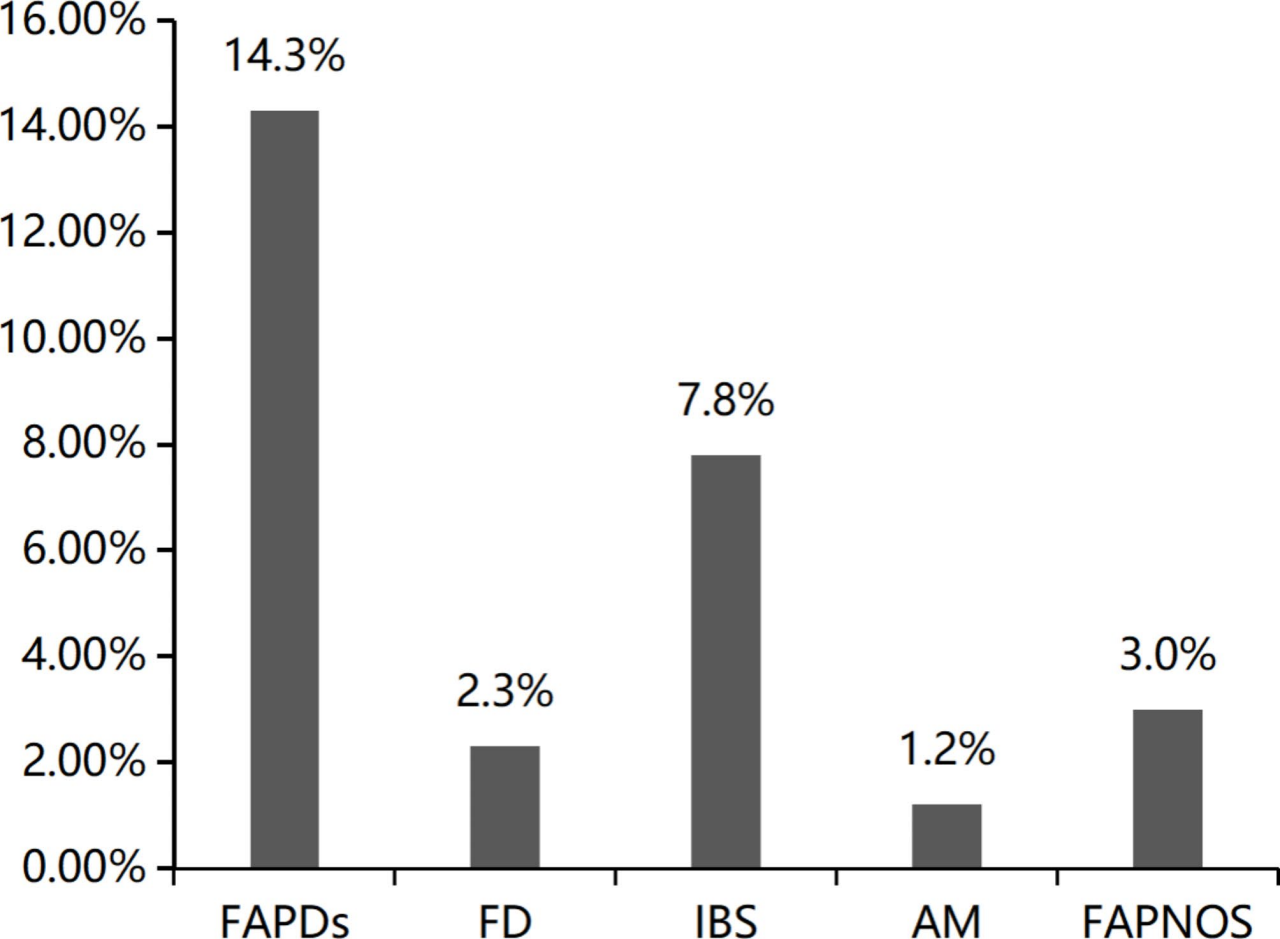
Prevalence of FAPDs and FAPD subtypes in southern Anhui

According to the Rome IV diagnostic criteria, among the 2,344 participants, 2,009 were free of FAPDs; the non-FAPD group had a mean age of 12.4 ± 3.0 years and a body mass index (BMI) of 20.9 ± 4.5 kg/m^2^, and 921 (45.8%) were girls. The other 335 (14.3%) children were diagnosed with FAPDs; the FAPD group had a mean age of 12.2 ± 3.2 years and a mean BMI of 20.7 ± 4.6 kg/m^2^, and 179 (53.4%) were girls. Of children with FAPDs, 55 (2.3%) had FD, 182 (7.8%) had IBS, 28 (1.2%) had AM, and 70 (3.0%) had FAPNOS. Thus, IBS was the most common subtype of FAPD in children in southern Anhui, and AM was the least common subtype. The prevalence of FAPDs was higher in females than in males (Figs. 1 and 2).

### Multivariate analyses of basic information

The study subjects were divided into three age groups: 6–9 years, 10–13 years and 14–17 years. We found no significant difference in the prevalence of FAPDs among the age groups (p = 0.488). The difference in BMI between the FAPD group and the non-FAPD group was also not significant (p = 0.415). We also found that although the prevalence of FAPDs was higher in girls than in boys, female sex was not a risk factor for FAPDs (OR = 1.208, 95% CI: 0.937–1.558, p = 0.145). However, the prevalence of FAPDs was lower in only children than in children with siblings, and being an only child was a protective factor against FAPDs (OR = 0.761, 95% CI: 0.589–0.984, p = 0.037) (Table 1).

**Table 1:** Univariate and multivariate analyses of demographic characteristics of participants in the FAPD and non-FAPD groups

Characteristic	Non-FAPD (n = 2,009)	FAPD (n = 335)	*P* ^1^	Multivariate analyses				
				OR	95% CI			*P* ^2^
**Sex**			0.010					
M	1088(54.2%)	156(46.6%)		1.00				
F	921(45.8%)	179(53.4%)		1.208		0.937–1.558	0.145	
**Age group**	12.4 ± 3.0	12.2 ± 3.2	0.488					
6–9 years	423(21.1%)	77(23.0%)						
10–13 years	668(33.3%)	104(31.0%)						
14–17 years	918(45.7%)	154(46.0%)						
**BMI (kg/m** ^**2**^ **)**	20.9 ± 4.5	20.7 ± 4.6	0.415					
**Only child status**			0.008					
Yes	1028(51.2%)	145(43.3%)		0.761		0.589–0.984	0.037	
No	981(48.8%)	190(56.7%)		1.00				

Values are expressed as the means ± SDs or n (%).

*P*^1^ value of univariate analyses; *P*^2^ value of multivariate analyses

### Academic stress exposure and FAPDs

In addition, we categorized respondents into four groups according to their academic performance: poor, average, good, and excellent. We found that there was no significant difference in the prevalence of FAPDs among the four academic performance groups (p = 0.129). Thus, academic performance was not a risk factor for FAPDs. In contrast, analysis of academic stress exposure showed that academic stress (OR = 1.452, 95% CI = 1.105–1.906, p = 0.007), academic performance below parental expectations (OR = 1.819, 95% CI = 1.629–2.066, p ≤ 0.001), and poor relationships with parents (OR = 2.067, 95% CI = 1.260–3.390, p = 0.004) were independent risk factors for FAPDs in children. Moreover, we classified children into three groups according to their sleep quality: normal sleep, difficulty falling asleep and early awakening. We found that sleep disorders, either difficulty falling asleep (OR = 2.665, 95% CI = 1.870–3.797, p ≤ 0.001) or early awakening (OR = 3.273, 95% CI = 1.792–5.977, p ≤ 0.001), were independent risk factors for FAPDs (Table 2).

**Table 2:** Univariate and multivariate analyses of academic stress in the FAPD and non-FAPD groups

Characteristic	Non-FAPD (n = 2,009)	FAPD (n = 335)	*P* ^1^	Multivariate analyses		
				OR	95% CI	*P* ^2^
**Academic performance**			0.129			
Poor	130(6.5%)	33(9.9%)				
Average	830(41.3%)	140(41.8%)				
Good	803(40.0%)	122(36.4%)				
Excellent	246(12.2%)	40(11.9%)				
**Academic stress**			< 0.001			
Yes	977(48.6%)	217(64.8%)		1.452	1.105–1.906	0.007
No	1032(51.4%)	118(35.2%)		1.00		
**Academic performance in relation to parental expectations**			< 0.001			
Lower	987(49.1%)	218(65.1%)		1.819	1.629–2.066	< 0.001
Equal	1022(50.9%)	117(34.9%)		1.00		
**Relationships with parents**			< 0.001			
Poor	58(2.9%)	37(11.0%)		2.067	1.260–3.390	0.004
Good	1951(97.1%)	298(89.0%)		1.00		
**Sleep disorder**			< 0.001			< 0.001
No sleep disorder	1829(91.0%)	247(73.7%)		1.00		
Difficulty falling asleep	133(6.6%)	70(20.9%)		2.665	1.870–3.797	< 0.001
Early awakening	47(2.3%)	18(5.4%)		3.273	1.792–5.977	< 0.001

Values are expressed as the means ± SDs or n (%)

Lower = lower than parents’ expectations; Equal = in line with parents’ expectation

*P*^1^ value of univariate, *P*^2^ value of multivariate analyses

### Creation of the nomogram

**Fig. 3:**
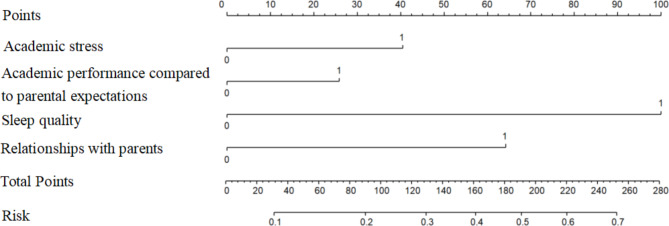
A nomogram for predicting the risk of functional abdominal pain disorders (FAPDs) in patients. The binary variables were coded as follows: academic stress (0 = no, 1 = yes), academic performance compared to parental expectations (0 = equal, 1 = lower than parents’ expectations), sleep quality (0 = no sleep disorder, 1 = sleep disorder (difficulty falling sleep or early awakening), and relationships with parents (0 = good, 1 = poor)

**Fig. 4:**
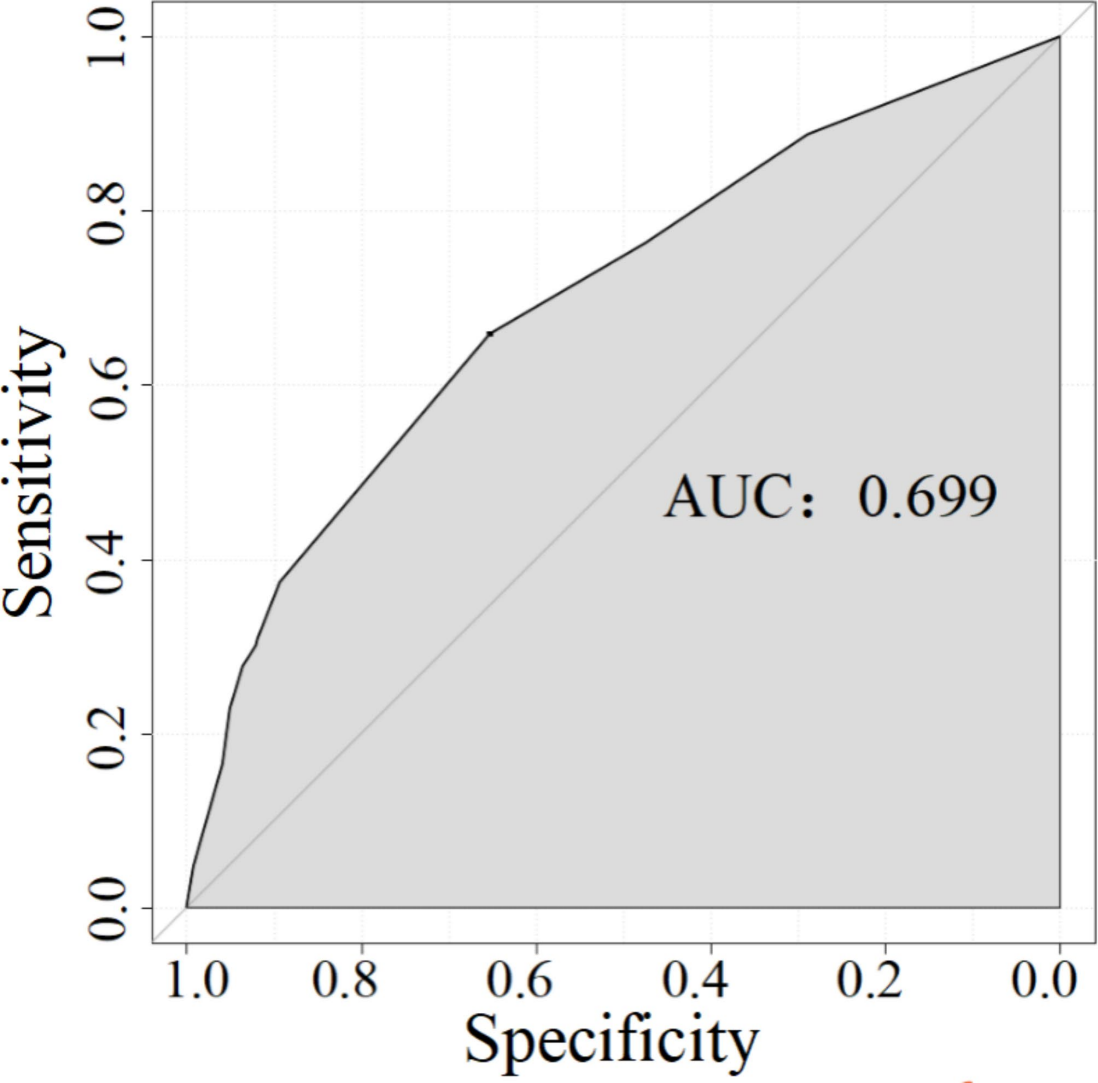
ROC curves of the nomogram

**Fig. 5:**
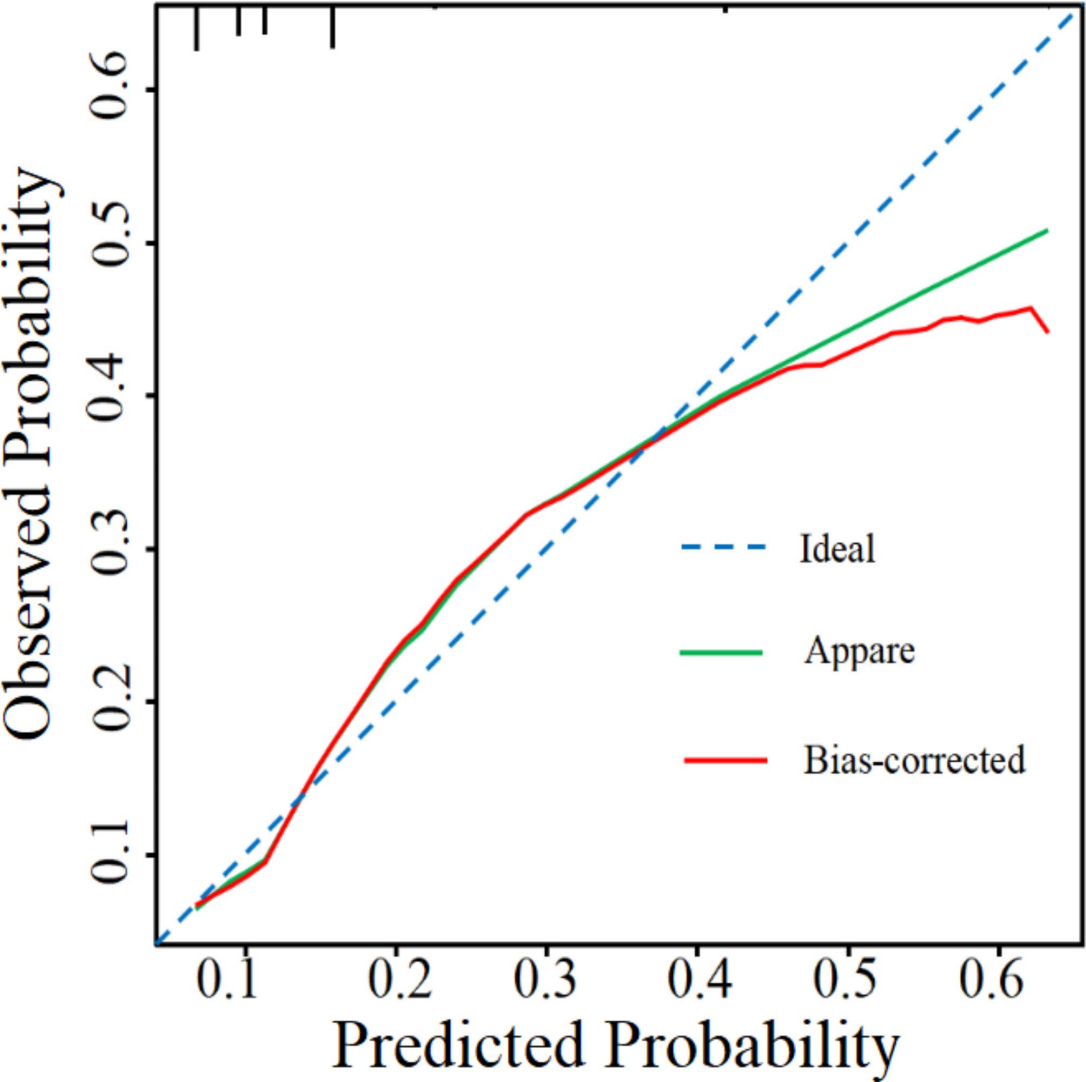
Calibration curves of the nomogram

We established a nomogram based on the independent risk factors for FAPDs (Fig. 3). The variables independently associated with FAPDs and included in the nomogram were academic stress, parental expectations, sleep disorders, and relationships with parents. A reference line at the top of the nomogram represents the score from 0 to 100 for each variable. The risk of FAPDs can be estimated effectively by summing the total score of each predictor. The predictive value of the nomogram was assessed by the ROC curve, calibration curve and Hosmer–Lemeshow test. The area under the curve (AUC) was 0.699, showing that the nomogram has good discrimination (Fig. 4). The calibration curve was close to the diagonal line, and the Hosmer–Lemeshow test was 0.342 (> 0.05) (Fig. 5).

## Discussion

FAPDs are one of the most common disorders in paediatric outpatient clinics and have a high prevalence worldwide [14–16]. Oswari et al. [17] reported that the prevalence of FAPDs in Indonesia among children aged 10–17 years was 11.5% in a study of 1,813 based on Rome III criteria; FD was the most common subtype, with a high prevalence in females. Devanarayana et al. [18] surveyed 2,163 children aged 10–16 years in Sri Lanka and found that the prevalence of FAPDs was 12.5%; IBS (prevalence: 4.9%) was the most common subtype, with a higher incidence rate in girls than in boys. They also found that the prevalence of FAPDs decreased with increasing age. A cross-sectional national study in Hungary [19] reported a prevalence of FAPDs of 11.9%, and AM was the most common subtype of FAPD, followed by IBS. In the meta-analysis by Korterink [20] FAPDs were reported to have a global prevalence of 13.5%, with IBS being the most common subtype (8.8%). They also found a higher prevalence of FAPDs in South America and Asia than in Europe and a higher prevalence in females than in males. This meta-analysis included a total of 58 clinical studies based on the Rome III criteria, including 196,472 children. Currently, reports of the prevalence of FAPDs and the most common subtypes vary according to countries and regions, and most studies have used the Rome III criteria, with relatively few studies using the Rome IV criteria. In the present study, we investigated the prevalence of FAPDs in children in southern Anhui, China using the Rome IV criteria. We found that the prevalence of FAPDs in southern Anhui was 14.3%, with a higher prevalence in girls than in boys. IBS was the most common subtype, followed by FAPNOS and FD; AM has the lowest prevalence. Although our research results are inconsistent with some of the above research results, thay are mainly consistent with the meta-analysis by Korterink [20].

At present, the risk factors for FAPDs remain unclear. Some studies have reported that infection, a history of early antibiotic use and changes in the gut microbiota are involved in the pathogenesis of FAPDs [21–23]. In addition, poor dietary habits such as excessive intake of fried foods, sweets, and stimulating foods as well as dairy allergies [24–26] are associated with the development of FAPDs. With the transformation of the medical model from the traditional “biomedical model” to the “biopsychosocial model”, increasing attention has been given to the relationship between the social environment and FAPDs in children and adolescents [27]. Studies have shown that the family environment, psychological factors, anxiety and depression are also associated with the development of FAPDs in children and adolescents [2, 17, 28, 29]. Zeevenhooven et al. [30] found that paternal dysphoria (but not maternal dysphoria) was associated with the development of FAPDs in children.

A survey [31] of 100 children with FAPDs found that the psychosocial factors associated with the onset of FAPDs included female sex, academic burden, poor financial status, examination stress, and school bullying. The relationship between psychosocial factors and FAPDs is unclear and controversial; clarifying the risk factors for FAPDs will establish a basis for their prevention and treatment. Major et al. [19] found that poor academic performance was a risk factor for FAPDs; however, our results are not consistent with that finding. We divided children into four groups according to their academic performance: poor, average, good, and excellent. We found no significant difference in the prevalence of FAPDs among these academic-performance groups (p = 0.129); therefore, we believe that academic performance is not a risk factor for FAPDs. Upon further investigation, we found that academic stress, academic performance lower than parental expectations, poor relationships with parents, and sleep disorders (difficulty falling asleep or early awakening) were independent risk factors for FAPDs in children. Thus, we concluded that academic stress exposure, not academic performance, is associated with the development of FAPDs. Furthermore, although the prevalence of FAPDs was higher in girls than in boys (p = 0.010), our regression analysis revealed that female sex was not a risk factor for FAPDs (OR = 1.208, 95% CI: 0.937–1.558, p = 0.145). In recent years, some studies [32, 33] have reported that obese children are more likely to develop FAPDs than normal-weight children, and obesity is considered an independent risk factor for FAPDs. In contrast, we found that BMI did not significantly differ between the FAPD and non-FAPD groups, suggesting that FAPDs may not be related to body weight in children in southern Anhui Province.

Currently, many Chinese parents force children to attend an excessive number of tutorial classes, resulting in psychological exhaustion [34]. Children’s frustration with excessive academic expectations is often not understood by their parents, leading children to experience negative emotions. Such inflated parental expectations appear unachievable to children, leading to hopelessness and physiological symptoms such as insomnia and abdominal discomfort. In addition, poor family relationships, especially poor relationships with parents, can lead to low self-esteem, withdrawn personalities and insecurity in children and adolescents, which can cause FGIDs [17, 29]. In the future, the treatment of psychological and physiological symptoms in children with FAPDs merits further attention.

The primary strength of our study is that it was a large cross-sectional study designed to investigate the prevalence of FAPDs (based on the Rome IV criteria) in children in southern Anhui Province, China and the association between FAPDs and academic stress.

### Limitations of the study

This study also has some limitations. First, our study population was recruited from schools in southern Anhui, which may limit the generalizability of these findings due to potential regional differences between our study population and children from other provinces in China. Therefore, our findings may not reflect the reality of the child population in China.We plan to conduct a multiregional study in the future to learn the role of academic stress in children with FAPDs all over China. Our study is useful in educating parents not to underestimate their role in their children’s health, and we will conduct studies to develop guidelines on family education and assess the positive effects on symptoms after parents change their behaviours.

## Conclusions

The prevalence of FAPDs in children in southern Anhui was similar to that reported worldwide, with a higher prevalence in girls than in boys; IBS was the most common subtype of FAPDs. In addition, academic stress (but not academic performance) was a risk factor for FAPDs in children in southern Anhui. Specifically, independent risk factors for FAPDs included academic stress, academic performance lower than parental expectations, and poor relationships with parents. Treatment of FAPDs in children therefore requires examining not only the physical symptoms but also the family environment and mental health of the children. Only by addressing these concerns will children receive the best treatment.

## Data Availability

The datasets used and/or analysed during the current study are available from the corresponding author upon valid request.

## Abbreviations

FAPDs: Functional abdominal pain disorders
IBS: Irritable bowel syndrome
FAPNOS: Functional abdominal pain–not otherwise specified
FD: Functional dyspepsia
AM: Abdominal migraine
FGIDs: Functional gastrointestinal disorders
ROC: Receiver-operating characteristic
BMI: Body mass index
